# AI-assisted 3D thoracic body composition analysis identifies candidate imaging markers associated with overall survival in older patients with lung cancer receiving radiotherapy

**DOI:** 10.3389/fonc.2026.1946212

**Published:** 2026-09-29

**Authors:** Saerom Sung, Hyun Joon Choi, Sangwoon Jeong, Wonyoung Cho, Yoon Jo Kim, Hyejung Cha, Chul Hee Min, Mi Sun Kim

**Affiliations:** 1 Department of Radiation Convergence Engineering, Yonsei University, Wonju, Republic of Korea; 2 Department of Radiation Oncology, Yonsei University Wonju College of Medicine, Wonju, Republic of Korea; 3 Oncosoft Inc., Seoul, Republic of Korea

**Keywords:** artificial intelligence, body composition, intermuscular fat, lung cancer, prognostic imaging markers, radiotherapy, simulation CT, skeletal muscle

## Abstract

**Background:**

Older patients with lung cancer receiving thoracic radiotherapy have heterogeneous outcomes that are not fully captured by conventional clinical descriptors. We evaluated whether artificial intelligence (AI)-assisted three-dimensional (3D) thoracic body-composition analysis of pretreatment radiotherapy simulation computed tomography (CT) could identify candidate body-composition imaging markers associated with overall survival.

**Methods:**

This exploratory retrospective single-institution study included 88 patients aged 70 years or older who received thoracic radiotherapy for lung cancer between 2019 and 2021. Thoracic skeletal muscle and intermuscular fat were segmented using an AI-assisted workflow followed by expert review and Hounsfield unit-constrained refinement. Twelve knowledge-informed candidate groups were evaluated using conventional Cox regression. After marker selection, the selected marker pair was assessed in continuous form using bootstrap optimism correction, repeated five-fold cross-validation, and Uno’s C-index. Penalized and alternative model-family analysis were performed as exploratory sensitivity analysis.

**Results:**

During follow-up, 28 deaths occurred. In an 18-case internal technical validation subset, the raw AI contours achieved Dice similarity coefficients of 0.930 for thoracic skeletal muscle and 0.723 for intermuscular fat. For Group 12, intermuscular adiposity index (IAI) and attenuation-weighted thoracic muscle fraction (TMF) were the variables that met the univariable screening criterion; the resulting two-marker model met the discrimination threshold without problematic collinearity. When modeled together, IAI values above the cohort median (hazard ratio [HR], 2.78; 95% confidence interval [CI], 1.25–6.18; P = 0.012) and TMF values above the cohort median (HR, 3.77; 95% CI, 1.52–9.32; P = 0.004) were associated with poorer overall survival. In continuous form, the selected marker pair had an optimism-corrected Harrell’s C-index of 0.751 (95% CI, 0.661–0.849), a mean repeated cross-validation C-index of 0.753 ± 0.112, and Uno’s C-index of 0.737. These performance estimates were conditional on prior marker selection.

**Conclusion:**

AI-assisted analysis of routine radiotherapy simulation CT identified IAI and TMF as candidate body-composition imaging markers associated with overall survival in this clinically heterogeneous cohort. The findings are exploratory and require independent evaluation in larger, clinically characterized cohorts before clinical use.

## 1 Introduction

Lung cancer remains a leading cause of cancer-related morbidity and mortality among older adults (1–4). In this population, treatment outcomes are influenced not only by tumor stage, histology, and treatment intensity, but also by host-related factors such as comorbidity, physiologic reserve, immune and metabolic changes, nutritional status, and functional vulnerability (5–8). These factors are particularly relevant for older patients receiving radiotherapy (RT), in whom pretreatment risk assessment may guide treatment planning, supportive care, and follow-up strategies. However, conventional clinical descriptors do not fully capture the heterogeneity of physiologic reserve in older patients with lung cancer.

Body mass index (BMI) is widely used as a simple marker of body habitus and has been associated with cancer outcomes (9, 10). Nevertheless, BMI cannot distinguish between skeletal muscle, adipose tissue, ectopic fat deposition, and muscle quality. This limitation is clinically important because skeletal muscle depletion, adipose infiltration, and altered body composition have been associated with survival, treatment tolerance, and adverse outcomes in patients with cancer, including lung cancer (11–14). Single-slice computed tomography (CT)-based body-composition analysis has provided important prognostic information, but it may not fully represent the three-dimensional (3D) distribution of thoracic muscle and intermuscular fat in patients undergoing thoracic RT (13, 15, 16).

RT simulation CT is routinely acquired before treatment and therefore represents an underused source of quantitative host-related information. Recent advances in automated segmentation and 3D CT analysis have enabled efficient volumetric assessment of skeletal muscle and adipose compartments (16–19). In the RT workflow, such approaches may allow opportunistic body-composition analysis without additional imaging or substantial manual contouring burden. However, the clinical utility of artificial intelligence (AI)-assisted 3D thoracic body-composition analysis using simulation CT remains insufficiently defined in older patients with lung cancer receiving RT.

Biomarker development from body-composition data also presents an analytic challenge. Many derived indices share anatomical measurements, normalization factors, or mathematical components, creating redundancy and multicollinearity that may inflate apparent performance and obscure interpretation. In a cohort with a limited number of outcome events, unrestricted selection among correlated candidate variables can further increase model instability and selection-related optimism. We therefore used a staged, knowledge-informed grouping strategy to reduce redundancy among mathematically related indices and preserve interpretability (20). Conventional survival analysis can be used to identify interpretable candidate markers, whereas secondary penalized and machine-learning approaches can examine the consistency of the candidate-group pattern and characterize the performance of selected markers under alternative modeling approaches.

We evaluated whether AI-assisted 3D thoracic body-composition analysis of routine pretreatment RT simulation CT could identify candidate imaging markers associated with overall survival in older patients with lung cancer. Conventional Cox regression across the knowledge-informed candidate groups served as the primary candidate-marker analysis. Secondary analysis were performed separately to examine whether the group-level pattern was retained under penalized modeling, to assess the internal performance of the selected marker pair after selection, and to determine whether the findings were materially dependent on the choice of survival-modeling approach.

## 2 Materials and methods

### 2.1 Study design, patients and outcome

This retrospective single-institution cohort study included older patients with lung cancer who received thoracic RT between 2019 and 2021. The study was approved by the Institutional Review Board of Yonsei University Wonju Severance Christian Hospital (IRB No. CR324037), and the requirement for informed consent was waived because of the retrospective nature of the study.

Patients were eligible if they were aged 70 years or older, received RT to the thoracic region for lung cancer, and had available RT simulation CT images and survival follow-up data. Patients younger than 70 years, those who received RT to non-thoracic sites, and those without analyzable simulation CT images or required clinical data were excluded. Demographic and clinical variables, including age, sex, body mass index (BMI), pathology, clinical stage, total radiation dose, completion of planned RT, follow-up duration, and survival status, were collected from the institutional registry and medical records. The primary outcome was overall survival, defined as the time from the start date of RT to death from any cause. Patients who were alive at the last follow-up were censored on the date of last known contact.

### 2.2 CT-based body-composition analysis and AI-assisted segmentation

Pretreatment RT simulation CT images were used for 3D thoracic body-composition analysis. The region of interest encompassed the thoracic muscle compartment from the sternoclavicular junction to the T12 vertebral level. Within the delineated outer muscle boundary, skeletal muscle and intermuscular fat (IMF) were identified using attenuation ranges of −29 to +150 HU and −190 to −30 HU, respectively, with manual correction of anatomical boundaries when required.

A smart-ensemble auto-segmentation model was developed using the OncoStudio training toolkit (Oncosoft Inc., Seoul, Republic of Korea) and nnU-Net-based 2D and 3D networks (21). The model was trained in 70 cases and technically evaluated in a separate 18-case validation set drawn from the same analytic cohort. Therefore, segmentation performance represented internal technical validation. In the validation set, raw AI-generated contours were compared with reference contours delineated by two medical physicists and reviewed by two radiation oncologists. Performance was assessed using the Dice similarity coefficient (DSC), 95% Hausdorff distance (HD95), sensitivity, specificity, positive predictive value, negative predictive value, and accuracy (22, 23). All AI-generated contours were subsequently reviewed and refined using MIM software version 7.1.8 (MIM Software Inc., Cleveland, OH, United States) before quantitative analysis.

From the final contours, mean attenuation and volume were measured separately for skeletal muscle and IMF. Tissue mass was estimated using a CT-based density calibration obtained with an advanced electron density phantom (Sun Nuclear Corporation, Melbourne, FL, United States), consistent with Task Group 66 CT simulation quality-assurance principles (24). Derived body-composition indices represented muscle quantity, muscle attenuation, intermuscular adiposity, and attenuation-weighted thoracic composition. Definitions, formulas, and units for all candidate indices are provided in **Supplementary Method 1**.

### 2.3 Candidate-group construction and primary Cox analysis

Several derived body-composition indices shared underlying anatomical measurements, normalization terms, or mathematical components. Simultaneous inclusion of closely related indices in a single multivariable model could therefore introduce substantial redundancy and multicollinearity. To limit this overlap, the candidate indices were organized into 12 candidate groups, with age and total radiation dose retained as common clinical covariates across all groups. The composition of each candidate group is provided in **Supplementary Table 1**.

Conventional Cox regression constituted the primary candidate-marker analysis. Within each group, imaging-derived indices were dichotomized at the cohort median and evaluated using univariable Cox proportional hazards models. Variables with P < 0.05 were entered into the corresponding group-wise multivariable Cox model. Group-level screening considered both model discrimination and multicollinearity. Harrell’s C-index was used to assess discrimination, with a threshold of 0.70 used as a screening criterion. Multicollinearity was assessed using Spearman’s rank correlation coefficient (ρ) and the variance inflation factor (VIF), with potentially problematic multicollinearity defined as |ρ| ≥ 0.70 or VIF > 5. Detailed group compositions and screening results are provided in **Supplementary Table 1**.

### 2.4 Post-selection internal performance assessment and descriptive survival grouping

After completion of the primary Cox-based screening analysis, the selected marker pair was subsequently evaluated as continuous predictors. We assessed the internal performance of the selected two-marker Cox model using 1,000 bootstrap resamples, repeated five-fold cross-validation with 100 repeats. We also calculated Uno’s C-index (25) to assess discrimination while accounting for censoring. Harrell’s C-index was used as the primary measure of discrimination. We performed resampling after the primary analysis had selected the marker pair and with the selected predictor set fixed during resampling. These estimates therefore describe the internal performance of the selected two-marker model, not the performance of the complete marker-selection process. For descriptive survival visualization, a model-derived score was calculated from the median-dichotomized selected markers, with each indicator weighted by its estimated Cox regression coefficient. Patients were divided at the cohort median score, and Kaplan–Meier curves with the log-rank test were used to compare the resulting groups.

### 2.5 Exploratory penalized and model-family sensitivity analysis

As a secondary group-level sensitivity analysis, we applied elastic-net Cox regression (26) to the same 12 candidate groups. We entered all variables within each group as continuous predictors and retained the full group structure. For each group, we assessed discrimination using Harrell’s C-index and applied bootstrap-based optimism correction. We used this analysis to examine whether the group-level discrimination pattern observed in the primary Cox analysis remained similar under penalized regression. We did not use the penalized analysis to change the marker selection from the primary Cox analysis.

For an exploratory reference comparison, we constructed a limited clinical model using age, BMI, and total radiation dose. We then added each selected marker separately and both markers together. We compared model discrimination using Harrell’s C-index and also reported the integrated Brier score and the 12-month time-dependent area under the receiver operating characteristic curve. We reported events per variable to show model complexity relative to the number of observed events. The reference model included only these three clinical variables and therefore served as an exploratory comparator.

We then evaluated the selected marker pair using elastic-net Cox regression, random survival forest (27), and gradient boosting survival models (28). We compared the discrimination of the selected marker pair across the three model families using repeated cross-validation. We used this comparison to determine whether its performance changed materially with the choice of survival model, not to select additional predictors.

We summarized continuous variables as medians and interquartile ranges and categorical variables as counts and percentages. All tests were two-sided, and P < 0.05 was considered statistically significant. We performed conventional survival analysis using SAS version 9.4 (SAS Institute Inc., Cary, NC, United States) and exploratory penalized and machine-learning analysis using Python version 3.10. **Figure 1** summarizes the study workflow from AI-assisted segmentation through primary Cox screening and secondary sensitivity analysis.

**Figure 1:**
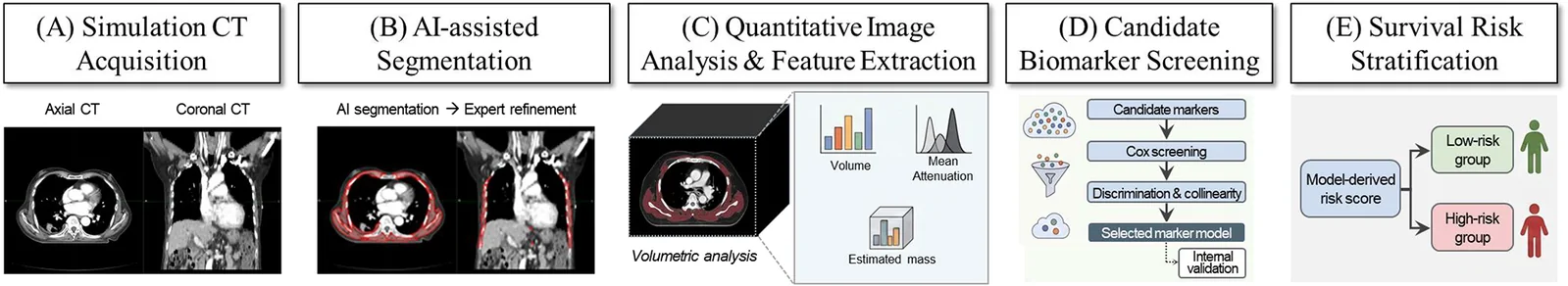
Overview of the AI-assisted three-dimensional thoracic body-composition and prognostic analysis workflow. **(A)** Acquisition of pretreatment radiotherapy simulation computed tomography images. **(B)** Artificial intelligence-assisted segmentation of thoracic skeletal muscle and intermuscular fat, followed by expert review and Hounsfield unit-constrained refinement. **(C)** Quantitative image analysis and feature extraction, including volume, mean attenuation, and estimated tissue mass. **(D)** Cox-based candidate marker screening, discrimination and collinearity assessment, marker-model selection, and internal performance assessment. **(E)** Model-derived score and descriptive survival grouping. AI, artificial intelligence; CT, computed tomography; HU, Hounsfield unit; IMF, intermuscular fat; RT, radiotherapy.

## 3 Results

### 3.1 Patient and treatment characteristics

The final analytic cohort included 88 older patients with lung cancer who received thoracic RT. The median age was 77 years, and 76 patients (86.4%) were male. The median BMI was 23.3 kg/m^2^, and the median total radiation dose was 6000 cGy. The cohort included 46 patients with squamous cell carcinoma, 18 with adenocarcinoma, 19 with small cell lung cancer, and 5 with other histologies. During follow-up, 28 patients died (31.8%), and 60 were censored. The median follow-up duration was 18.7 months. Among the patients who died, the median time from RT initiation to death was 10.3 months. **Table 1** summarizes the patient and treatment characteristics.

**Table 1:** Patient and treatment characteristics of the analytic cohort (N = 88).

Characteristic	Overall cohort (N = 88)
Age, years	
Median (IQR)	77 (74–80)
Sex	
Male	76 (86.4)
Female	12 (13.6)
Body mass index, kg/m^2^	
Median (IQR)	23.3 (20.8–25.4)
Pathology	
Squamous cell carcinoma	46 (52.3)
Adenocarcinoma	18 (20.5)
Small cell lung cancer	19 (21.6)
Other	5 (5.7)
Clinical stage: non-small cell lung cancer	
Stage I	12 (13.6)
Stage II	28 (31.8)
Stage III	7 (8.0)
Stage IV	12 (13.6)
Not available	10 (11.4)
Clinical stage: small cell lung cancer	
Limited stage	2 (2.3)
Extensive stage	1 (1.1)
Not available	16 (18.2)
Total radiation dose, cGy	
Median (IQR)	6000 (5000–6030)
Completion of planned radiotherapy	
No	12 (13.6)
Yes	76 (86.4)
Death during follow-up	
No	60 (68.2)
Yes	28 (31.8)
Follow-up duration, months	
Median (IQR)	18.7 (6.9–45.1)
Survival duration from radiotherapy start, months	
Median (IQR)	10.3 (5.1–23.9)

Data are presented as n (%) unless otherwise indicated. Continuous variables are presented as median (interquartile range). Survival duration from the start of radiotherapy is summarized among patients who died during follow-up (n = 28).

### 3.2 AI-assisted segmentation performance

Before expert refinement, the raw AI-generated contours yielded DSC values of 0.913 for the combined thoracic muscle–intermuscular fat volume, 0.930 for thoracic skeletal muscle, and 0.723 for intermuscular fat. The corresponding HD95 values were 3.947, 3.200, and 8.196 mm, respectively. All contours were subsequently reviewed and refined before quantitative feature extraction. Sensitivity, specificity, positive predictive value, negative predictive value, and accuracy are reported in **Supplementary Table 2**.

### 3.3 Primary Cox analysis and marker selection

Among the 12 candidate groups, Group 8 met the C-index screening threshold (0.764) but exceeded both collinearity thresholds (maximum |ρ| = 0.970; maximum VIF = 12.32). Group 12 also met the C-index threshold (0.724) and remained below both collinearity thresholds (maximum |ρ| = 0.450; maximum VIF = 1.48). We therefore selected Group 12 for the primary Cox analysis.

Within Group 12, intermuscular adiposity index (IAI) and attenuation-weighted thoracic muscle fraction (TMF) met the univariable screening criterion and were entered into the multivariable Cox model. When modeled together, IAI values above the cohort median were associated with a higher hazard of death (HR, 2.78; 95% CI, 1.25–6.18; P = 0.012), as were TMF values above the cohort median (HR, 3.77; 95% CI, 1.52–9.32; P = 0.004) (**Table 2**). The cohort medians used for dichotomization were −18,961.83 HU for IAI and 1,521.73 HU·mL/kg for TMF.

**Table 2:** Univariable and multivariable Cox regression analyses of variables in the selected Group 12 candidate set.

Variable	Univariable HR (95% CI)	P-value	Multivariable HR (95% CI)	P-value
Age (years)	1.03 (0.99–1.08)	0.124	—	—
Total dose (cGy)	1.00 (0.99–1.01)	0.571	—	—
BMI (kg/m**^2^**)	0.98 (0.92–1.04)	0.411	—	—
IAI	2.47 (1.11-5.49)	0.027	2.78 (1.25–6.18)	0.012
TMF	3.40 (1.38-8.38)	0.008	3.77 (1.52–9.32)	0.004

BMI, body mass index; CI, confidence interval; HR, hazard ratio; IAI, intermuscular adiposity index; TMF, attenuation-weighted thoracic muscle fraction.

Age, total radiotherapy dose, and BMI were modeled as continuous variables. IAI and TMF were dichotomized at their cohort-specific medians of -18,961.83 and 1,521.73, respectively. Hazard ratios for IAI and TMF compare values above versus at or below the corresponding median. Variables meeting the screening criterion were entered into the multivariable model.

### 3.4 Post-selection performance and descriptive survival analysis

After IAI and TMF were selected in the primary Cox analysis, they were evaluated as continuous predictors. The optimism-corrected Harrell’s C-index was 0.751 (95% CI, 0.661–0.849), and repeated five-fold cross-validation yielded a mean C-index of 0.753 ± 0.112. Uno’s C-index was 0.737. **Table 3** provides the complete performance estimates, including the apparent C-index and the corresponding results for the median-dichotomized model. To describe survival differences across the model-derived score, patients were divided into lower- and higher-score groups using the cohort median as the cutoff (n = 44 each). The higher-score group had poorer overall survival than the lower-score group (log-rank P = 0.0048; **Figure 2**).

**Table 3:** Post-selection internal performance of Cox models containing continuous or median-dichotomized IAI and TMF.

Model	C-index	Optimism-corrected C-index (95% CI)	cross-validation C-index	Uno’s C-index
Continuous IAI + TMF	0.764	0.751 (0.661–0.849)	0.753 ± 0.112	0.737
Dichotomized IAI + TMF	0.724	0.709 (0.618–0.804)	0.694 ± 0.097	0.670

The apparent C-index was estimated in the full analytic cohort. Optimism-corrected C-indices and 95% confidence intervals were obtained from 1,000 bootstrap resamples. Cross-validation results are presented as mean ± standard deviation from five-fold cross-validation repeated 100 times. Performance estimates were calculated after IAI and TMF had been selected in the primary Cox analysis and are therefore conditional on prior marker selection.

**Figure 2:**
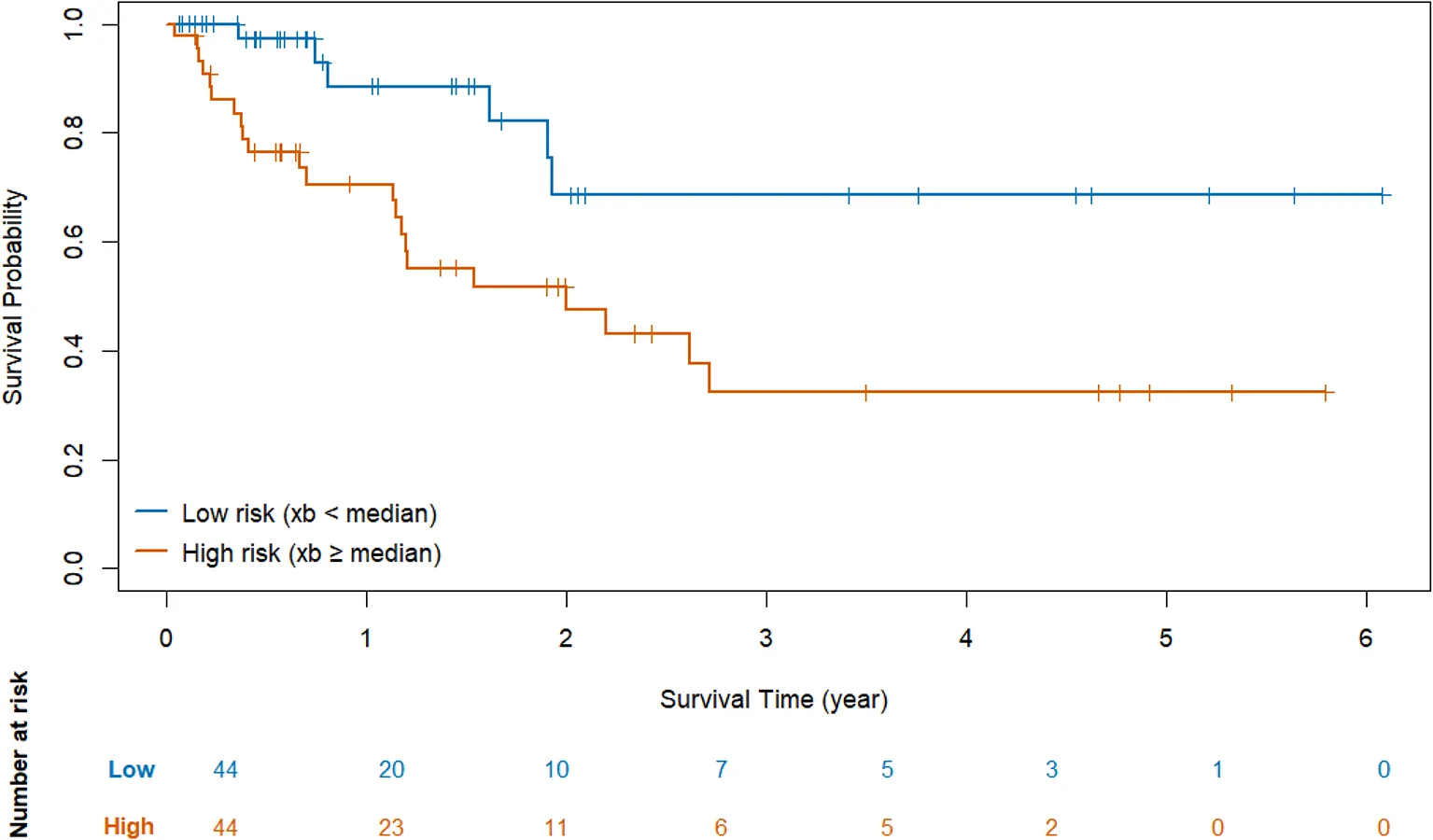
Overall survival according to the Cox model–derived score. Kaplan–Meier estimates are shown for the lower model-score group (n = 44; 6 deaths) and the higher model-score group (n = 44; 22 deaths). Groups were defined using the cohort median model score.

### 3.5 Exploratory sensitivity analysis

As a secondary group-level sensitivity analysis, we evaluated the same 12 candidate groups using elastic-net Cox regression. Optimism-corrected C-indices ranged from 0.607 to 0.762. Groups 8 and 12 had the highest corrected C-indices (both 0.762) (**Supplementary Table 3**). In this analysis, Group 12 retained all five original variables—age, total radiation dose, BMI, IAI, and TMF—and therefore differed from the selected two-marker IAI–TMF model.

The same IAI–TMF predictor set was evaluated using elastic-net Cox regression, random survival forest, and gradient boosting survival. The mean cross-validated C-indices were 0.751 ± 0.112 for elastic-net Cox, 0.714 ± 0.106 for random survival forest, and 0.665 ± 0.110 for gradient boosting survival (**Supplementary Table 4**).

An exploratory reference-model comparison included a limited clinical reference model comprising age, BMI, and total radiation dose; the same clinical model with IAI and TMF added; and a model containing only IAI and TMF. The limited clinical reference model had an optimism-corrected C-index of 0.582. Adding IAI and TMF to the same clinical covariates yielded a corrected C-index of 0.762, whereas the model containing only IAI and TMF had a corrected C-index of 0.751. No formal paired comparison between these models was performed. **Table 4** provides the corresponding integrated Brier scores and 12-month time-dependent AUCs.

**Table 4:** Exploratory performance of a limited clinical reference model and imaging-containing elastic-net Cox models.

Model	Optimism-corrected C-index (95% CI)	EPV	IBS	12-month AUC
Clinical	0.582 (0.452–0.685)	9.3	0.174	0.576
Clinical + IAI	0.628 (0.504–0.733)	7.0	0.175	0.637
Clinical + TMF	0.729 (0.623–0.814)	7.0	0.155	0.780
Clinical + IAI + TMF	0.762 (0.664–0.842)	5.6	0.155	0.810
IAI + TMF	0.751 (0.661–0.849)	14.0	0.155	0.771
IAI	0.570 (0.449–0.689)	28.0	0.171	0.588
TMF	0.695 (0.603–0.783)	28.0	0.160	0.676

AUC, area under the time-dependent receiver operating characteristic curve; EPV, events per variable; IAI, intermuscular adiposity index; IBS, integrated Brier score; TMF, attenuation-weighted thoracic muscle fraction.

The clinical model included age, BMI, and total radiotherapy dose. Optimism-corrected C-indices were obtained from 1,000 bootstrap resamples. IBS was calculated over 60–700 days, and time-dependent AUC was evaluated at 12 months. EPV was calculated as the number of events divided by the number of candidate predictors and does not represent the effective degrees of freedom after penalization.

## 4 Discussion

AI-assisted volumetric analysis of routine RT simulation CT identified IAI and TMF as candidate imaging markers associated with overall survival in this cohort. After IAI and TMF had been selected, the continuous two-marker model yielded C-index estimates ranging from 0.737 to 0.753 across the post-selection analysis. These analyses began only after the marker pair had been selected and therefore evaluated the selected IAI–TMF model, not the preceding marker-selection process.

The primary analysis considered both discrimination and multicollinearity because several candidate indices shared anatomical measurements, normalization terms, or mathematical components. Group 8 met the C-index screening threshold but showed substantial multicollinearity. Group 12 met the same threshold with substantially lower collinearity and was therefore selected for the primary analysis. Within Group 12, IAI and TMF met the univariable screening criterion and were subsequently included in the two-marker multivariable Cox model. Dichotomizing IAI and TMF at their cohort medians simplified the initial screening and descriptive survival grouping, but it reduced the information available from the full range of continuous marker values. When IAI and TMF were analyzed as continuous variables, the optimism-corrected C-index was higher than that of the median-dichotomized model. Future prognostic studies should therefore also evaluate IAI and TMF as continuous measures.

The secondary analysis examined the primary findings using different survival-modeling approaches. At the group level, we re-evaluated the original 12 candidate groups using elastic-net Cox regression. Groups 8 and 12 again had the highest optimism-corrected C-indices. This secondary analysis was not used to change the marker selection from the primary Cox analysis. We then evaluated the same IAI–TMF predictor set using elastic-net Cox, random survival forest, and gradient boosting survival. Elastic-net Cox yielded a mean cross-validated C-index close to that of the conventional continuous Cox model, whereas random survival forest and gradient boosting survival yielded lower mean values. These model comparisons were used to examine the selected IAI–TMF pair across different survival models, not to select additional markers or identify a superior algorithm.

We also compared the selected imaging markers with a limited clinical reference model. The model containing age, BMI, and total radiation dose had an optimism-corrected C-index of 0.582, whereas adding IAI and TMF increased the corrected C-index numerically to 0.762. We did not formally test this difference, and the reference model did not include several established prognostic factors. The numerical increase, therefore, does not establish that IAI and TMF add prognostic value beyond established clinical factors. A more complete clinical model is needed to answer that question.

IAI and TMF describe different aspects of thoracic body composition that BMI does not capture. Previous studies in patients with lung cancer have linked CT-derived measures of skeletal muscle, adiposity, and body composition with survival and other clinical outcomes (9–14). Our results add to this literature by identifying two composite thoracic indices that were associated with overall survival in this cohort.

IAI combines intermuscular fat attenuation with the relative amount of intermuscular fat. Higher IAI was associated with poorer overall survival. Previous studies have linked greater intermuscular adiposity with impaired physical performance in older adults and with metabolic dysfunction (29–31). Skeletal muscle adiposity has also been associated with adverse cardiovascular outcomes (32). These observations provide biological context for the association between IAI and survival, although the present study did not directly measure physical function, metabolic status, or cardiovascular risk.

Higher TMF was also associated with poorer overall survival, but the biological basis of this association is less clear. TMF combines muscle attenuation, thoracic muscle volume, and body weight in a single measure. CT-based studies in lung cancer have evaluated muscle quantity and muscle attenuation as distinct components of body composition, and both have been associated with clinical outcomes (9, 10, 13, 14). In TMF, muscle attenuation provides information on the CT characteristics of muscle tissue, whereas thoracic muscle volume represents the amount of muscle included in the analyzed region. Normalization by body weight further expresses these muscle measurements relative to the patient’s overall body weight. For the same muscle attenuation and volume, a lower body weight would result in a higher TMF value. A higher TMF therefore does not directly indicate greater muscle quantity, higher muscle attenuation, or preserved muscle reserve. The observed association between higher TMF and poorer survival may reflect the combined contribution of muscle characteristics and body-weight context, but this explanation requires confirmation in independent cohorts.

Pretreatment simulation CT is already acquired for routine RT planning, so body-composition measurements can be obtained without additional imaging. Previous studies have shown that multi-slice or volumetric CT analysis can provide body-composition information beyond that obtained from a single axial slice (15–19). In the present study, the analysis extended from the sternoclavicular junction to T12 and assessed muscle and intermuscular fat across a broad thoracic volume.

Automated CT segmentation has increasingly been used for body-composition analysis and other quantitative imaging tasks, with growing interest in clinical implementation (13, 16–19, 33, 34). In our study, segmentation agreement was higher for skeletal muscle than for intermuscular fat. All final contours underwent expert review and HU-constrained refinement before quantitative feature extraction. The current workflow is therefore AI-assisted rather than fully automated. Its reproducibility across institutions, scanners, acquisition protocols, and contrast conditions remains to be established.

If independent cohorts confirm these associations, IAI and TMF could complement established clinical factors, such as tumor stage, performance status, and systemic therapy, in pretreatment assessment. Their potential clinical role may include identifying patients who warrant additional nutritional, functional, or geriatric assessment. The present data do not support the use of IAI or TMF for oncologic treatment selection.

This study has several limitations. It was retrospective and single-center and included 88 patients with 28 deaths. The cohort included multiple histologic subtypes and stages, and stage information was unavailable for 26 of 88 patients. Detailed information on performance status, comorbidities, systemic therapy, frailty, nutritional interventions, pulmonary function, and cause-specific mortality was incomplete. These factors limit the ability to separate the prognostic information provided by body composition from that provided by established clinical factors.

The segmentation and prognostic analysis also have technical limitations. The segmentation model was technically evaluated in an 18-case subset from the same analytic cohort, and all final contours underwent expert review and HU-constrained refinement. Its performance outside the study setting therefore remains unknown. Resampling began after IAI and TMF had already been selected, and the complete 12-group screening and marker-selection procedure was not repeated within each resample. The resampling results therefore do not show whether the same group and the same markers would be selected in new samples, and some selection-related optimism may remain. The clinical reference model included only a limited set of covariates, calibration and decision-curve analyses were not performed, and the model-derived grouping threshold was specific to this cohort. Independent multicenter studies are needed to evaluate IAI and TMF in clinically well-characterized cohorts, compare them with comprehensive clinical models, and determine whether the segmentation and marker-selection workflow can be reproduced.

## 5 Conclusion

AI-assisted volumetric analysis of routine RT simulation CT identified IAI and TMF as candidate body-composition imaging markers associated with overall survival in older patients with lung cancer. The selected two-marker model showed similar discrimination estimates across several post-selection internal assessments, supporting further investigation of simulation CT as a source of host-related prognostic information. External validation of the complete marker-selection and modeling process is required before clinical application.

## Data Availability

The datasets generated and analyzed during the current study are not publicly available because they contain potentially identifiable patient information and are subject to institutional and ethical restrictions. Requests to access the datasets should be directed to the corresponding author and will be considered subject to approval by the relevant institutional review board.
