# Supplementary material for: AI-assisted 3D thoracic body composition analysis identifies candidate imaging markers associated with overall survival in older patients with lung cancer receiving radiotherapy
Source: Front Oncol. 2026 Sep 29;16:1946212. doi: 10.3389/fonc.2026.1946212 (PMC13658111; doi:10.3389/fonc.2026.1946212)
Supplement: Supplementary file 1 [file DataSheet1.docx]

**Supplementary Method 1**. Definitions of CT-derived body-composition indices

- BMI (kg/m²): W_body_ / Height² (*Note: W_body_ refers to whole body weight.*)
- Muscle Mass Index (MMI, kg/m²): W_muscle_ / Height²
- IMF Mass Index (IMFMI, kg/m²): W_IMF_ / Height²
- Muscle Volume Index (MVI, A.U.): V_muscle_ / Height³
- IMF Volume Index (IMFVI, A.U.): V_IMF_ / Height³
- Intermuscular Adiposity Index (IAI, HU): IMFD / (IMFMI / BMI)
- Muscle-IMF Density Product (MIDP, HU^2^): MD × |IMFD|
- Muscle Quality Index (MQI, HU): MD / (MMI / BMI)
- Attenuation-weighted Thoracic Muscle Fraction (TMF, HU·mL/kg): MD × V_muscle_ / W_body_
- Attenuation-weighted Thoracic IMF Fraction (TIF, HU·mL/kg): IMFD × V_IMF_ / W_body_

**Supplementary Table 1**. Candidate feature groups, conventional Cox regression–based screening, and multicollinearity diagnostics

| Group | Candidate variables | C-index | Max \|ρ\| | Max VIF |
| --- | --- | --- | --- | --- |
| 1 | Age, total dose, BMI, IAI, MQI | NE | 0.410 | 1.65 |
| 2 | Age, total dose, IAI, MQI | NE | 0.410 | 1.28 |
| 3 | Age, total dose, TIF, TMF | 0.660 | 0.354 | 1.25 |
| 4 | Age, total dose, BMI, TIF, TMF | 0.660 | 0.469 | 1.66 |
| 5 | Age, total dose, BMI, IAI | 0.610 | 0.402 | 1.38 |
| 6 | Age, total dose, BMI, TIF, IAI, TMF, MQI | NE | 0.970 | 2.62 |
| 7 | Age, total dose, BMI, MIDP | NE | 0.306 | 1.17 |
| 8 | Age, total dose, BMI, TIF, IAI, TMF, MIDP, MQI | 0.764 | 0.970 | 12.32 |
| 9 | Age, total dose, BMI, MQI | 0.621 | 0.056 | 1.01 |
| 10 | Age, total dose, BMI, TMF | 0.660 | 0.450 | 1.38 |
| 11 | Age, total dose, BMI, TIF | NE | 0.469 | 1.54 |
| 12 | Age, total dose, BMI, IAI, TMF | 0.724 | 0.450 | 1.48 |

Candidate imaging indices were dichotomized at the cohort median. Variables with univariable Cox P < 0.05 were entered into group-wise multivariable Cox models. A Harrell’s C-index threshold of 0.70 was used for group screening. Potential multicollinearity was assessed using maximum absolute Spearman correlation and variance inflation factor. NE indicates that a multivariable C-index was not estimated because no variable passed univariable screening.

**Supplementary Table 2.** Segmentation performance of the AI-assisted model

| **ROI** | **DSC** | **HD95 (mm)** | **Sensitivity** | **Specificity** | **PPV** | **NPV** | **Accuracy** |
| --- | --- | --- | --- | --- | --- | --- | --- |
| Combined volume | 0.913 | 3.947 | 0.945 | 0.994 | 0.884 | 0.997 | 0.992 |
| Thoracic skeletal muscle | 0.930 | 3.200 | 0.967 | 0.995 | 0.897 | 0.998 | 0.994 |
| Intermuscular fat | 0.723 | 8.196 | 0.936 | 0.999 | 0.721 | 0.999 | 0.998 |

**Supplementary Table 3.** Exploratory bootstrap-corrected performance of the 12 candidate feature-group elastic-net Cox models

| **Group** | **C-index** | **Optimism-corrected**  **C-index (95% CI)** | **Optimism** |
| --- | --- | --- | --- |
| 1 | 0.703 | 0.639 (0.522-0.732) | 0.064 |
| 2 | 0.689 | 0.635 (0.518-0.729) | 0.054 |
| 3 | 0.799 | 0.756 (0.654-0.836) | 0.043 |
| 4 | 0.802 | 0.749 (0.641-0.832) | 0.053 |
| 5 | 0.691 | 0.628 (0.504-0.733) | 0.063 |
| 6 | 0.806 | 0.749 (0.646-0.829) | 0.057 |
| 7 | 0.761 | 0.718 (0.610-0.809) | 0.043 |
| 8 | 0.814 | 0.762 (0.669-0.839) | 0.052 |
| 9 | 0.700 | 0.641 (0.521-0.737) | 0.059 |
| 10 | 0.774 | 0.729 (0.623-0.814) | 0.045 |
| 11 | 0.674 | 0.607 (0.479-0.707) | 0.067 |
| 12 | 0.812 | 0.762 (0.664-0.842) | 0.05 |

**Note**: Group 12 denotes the unreduced five-variable model including age, total radiation dose, BMI, IAI, and TMF. It should not be confused with the reduced two-marker IAI–TMF model evaluated in the primary and secondary analyses.

**Supplementary Table 4.** Model-family sensitivity analyses using IAI and TMF

| **Model** | **Configuration** | **Cross-validated**  **C-index (mean ± SD)** | **95% range** |
| --- | --- | --- | --- |
| ElasticNet Cox | IAI + TMF | 0.751 ± 0.112 | 0.513-0.929 |
| Random Survival Forest | IAI + TMF | 0.714 ± 0.106 | 0.485-0.891 |
| Gradient Boosting Survival | IAI + TMF | 0.665 ± 0.110 | 0.445-0.868 |
